# Clinical significance of automatic warning function of cardiac remote monitoring systems in preventing acute cardiac episodes

**DOI:** 10.12669/pjms.306.5484

**Published:** 2014

**Authors:** Shou-Qiang Chen, Shan-Shan Xing, Hai-Qing Gao

**Affiliations:** 1 Shou-Qiang CHEN, MD, PhD, Department of Cardiology, the Second Affiliated Hospital, Shandong University of Traditional Chinese Medicine, Jinan 250001, China.; 2 Shan-Shan XING, MD, PhD, Hai-Qing GAO, MD, Shandong University of Traditional Chinese Medicine, Jinan 250355, China.; 3 Cardiac Remote Monitoring Center, QiLu Hospital, Shandong University, Jinan 250012, China.

**Keywords:** Cardiac remote monitoring system, Automatic warning function, Acute cardiac episodes, Earlier diagnosis, Prognosis

## Abstract

***Objective: ***In addition to ambulatory Holter electrocardiographic recording and transtelephonic electrocardiographic monitoring (TTM), a cardiac remote monitoring system can provide an automatic warning function through the general packet radio service (GPRS) network, enabling earlier diagnosis, treatment and improved outcome of cardiac diseases. The purpose of this study was to estimate its clinical significance in preventing acute cardiac episodes.

***Methods: ***Using 2 leads (V1 and V5 leads) and the automatic warning mode, 7160 patients were tested with a cardiac remote monitoring system from October 2004 to September 2007. If malignant arrhythmias or obvious ST-T changes appeared in the electrocardiogram records was automatically transferred to the monitoring center, the patient and his family members were informed, and the corresponding precautionary or therapeutic measures were implemented immediately.

***Results:*** In our study, 274 cases of malignant arrhythmia, including sinus standstill and ventricular tachycardia, and 43 cases of obvious ST-segment elevation were detected and treated. Because of early detection, there was no death or deformity.

***Conclusions:*** A cardiac remote monitoring system providing an automatic warning function can play an important role in preventing acute cardiac episodes.

## INTRODUCTION

Acute cardiac episodes, including acute coronary artery syndrome and malignant arrhythmia, are the leading causes of sudden cardiac death (SCD).^1^^,^^2^ Seventy percent of these patients do not receive the early diagnosis and treatment that would prevent death.^3^^,^^4^ The effective prevention of acute cardiac episodes has become an investigative focus among researchers all over the world.^5^^-^^8^ Following the development of ambulatory Holter electrocardiographic recording and transtelephonic electrocardiographic monitoring (TTM), cardiac remote monitoring systems were researched widely and then applied in clinical practice.^9^^-^^13^ However, ambulatory Holter electrocardiographic recording can only be used to analyze records retrospectively.^14^ TTM can provide real-time monitoring, but it is limited to a certain extent because it transfers electrocardiogram records through only an immobile telephone.^15^^,^^16^

A cardiac remote monitoring system, which overcomes the disadvantages of the instruments mentioned above, has an automatic warning function via GPRS. When electrocardiogram records exceed the automatic warning thresholds established beforehand, they are automatically transferred to the hospital’s monitoring center. If malignant arrhythmias or obvious ST-T changes appear in the electrocardiogram records, the patient and his family numbers are informed so that the corresponding measures can be implemented immediately. Thus, the mortality and deformity rates of cardiovascular disease can be decreased. The aim of this study was to evaluate the clinical significance of the automatic warning function of a cardiac remote monitoring system in preventing acute cardiac episodes.

## METHODS

***Clinical data:*** The study subjects were recruited from the Qilu Hospital of Shandong University, and all subjects gave written informed consent. We studied 7160 patients (56.31±14.76 years, ranging from 4 to 102 yrs) from October 2004 to September 2007, which included 4189 males (58.50%) and 2971 females (41.50%). Of these 7160 patients, 1136 cases had a past history of angina pectoris, 755 cases had a past history of myocardial infarction, 3410 cases had a past history of arrhythmia, 912 cases had a past history of hypertension, 226 cases were implanted with a pacemaker, and 721 cases had comorbid diseases.

**Fig.1:**
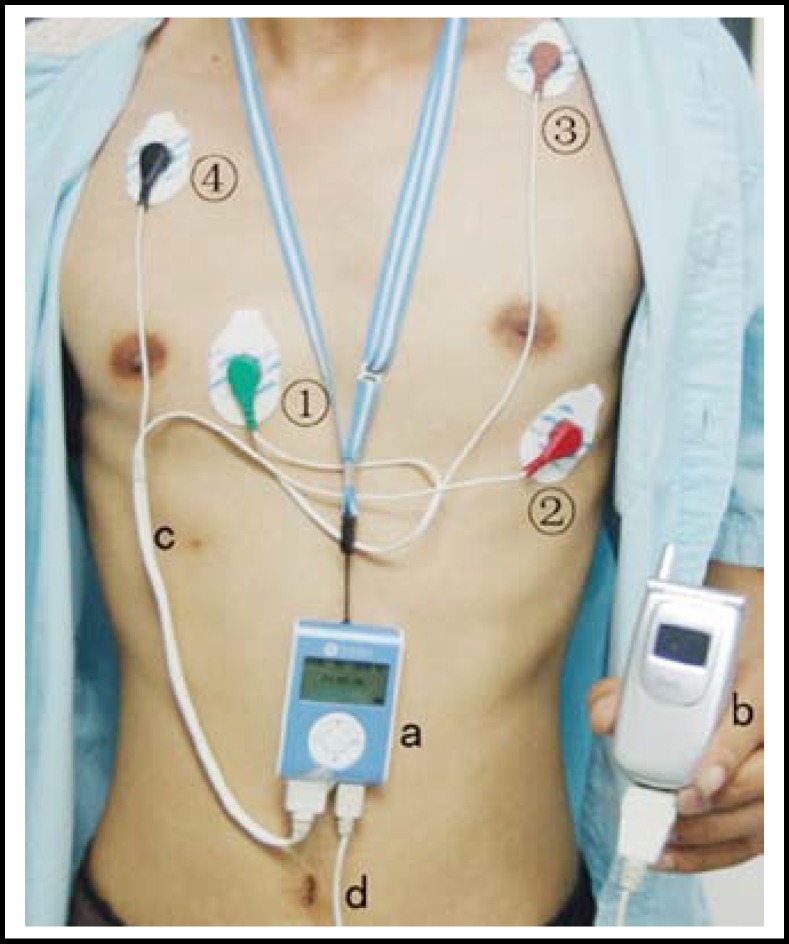
A cardiac remote monitor and its location.

***Instruments: ***A cardiac remote monitoring system involves a cardiac remote monitoring center established in the hospital and a cardiac remote monitor worn by the patient. The former, managing from fifty thousand to a hundred thousand patients at the same time, is composed of a database server, an application server, a network server and a doctor’s station. The latter, using 2 leads (V1 and V5 leads), is comprised of a pre-warning device, a special mobile telephone, a leads’ line and a data line (Fig.1).

A cardiac remote monitoring system is a kind of network based on Transmission Control Protocol/Internet Protocol (TCP/IP) for commuting and managing cardiac monitoring data. It has not only an ambulatory Holter electrocardiographic recording and a TTM function, but also an automatic warning function via self-adaptive analyzing and pre-warning software. The cardiac remote monitor records electrocardiographic data continuously. When the data exceed any threshold ordered beforehand, the cardiac remote monitor warns the doctor’s station immediately by activating a digital communication module, landing GPRS network, establishing a logistic connection, acquiring an IP address and passing validation. The automatic warning threshold of tachycardia is ≥135 beats per minute, that of bradycardia is ≤45 beats per minute, that of premature beat is ≥5 beats per minute, that of ST-segment depression is ≥1mm, that of ST-segment elevation is ≥1mm, that of standstill is ≥2s, and that of R- on-T is ≥1 beat.

***Methods: ***After a resting electrocardiogram examination and routine registration including name, sex, age, profession, address, telephone and medical records, all patients were given a cardiac remote monitor using an automatic warning mode. They were instructed how to use the device. They were told they could also use the manual recording mode with each occurrence of symptoms. The doctor on duty was responsible for diagnosing each electrocardiogram recording transferred to the monitoring center. If malignant arrhythmias or obvious ST-T changes appeared, the patient or his family members would be informed immediately, and the corresponding precautionary or therapeutic measures were implemented immediately. After the apparatus was taken off, the electrocardiogram data stored inside it would be imported into a computer through a universal serial bus (USB) connection and analyzed retrospectively to determine further treatment.

***Statistical analysis: ***Quantitative variables are presented as mean ± SD. For patients suffering from acute myocardial infarction and receiving percutaneous coronary intervention, the time of symptom-onset-to-door (SOTD), the time of door-to-consult (DTC), the time of consult-to-lab (CTL), the time of lab-to-balloon (LTB) and the time of symptom-onset-to-balloon (SOTB) were analyzed statistically and compared with those of Yang Xinchun’s study via *t*-test respectively.^17^ A probability value of *P*<0.05 was considered statistically significant. SPSS version 13.0 for Windows was used in analysis.

## RESULTS

Among the patients, the longest period of time for wearing the cardiac remote monitor was 65 days, while the shortest period was only one day. The maximum number of electrocardiogram records transferred automatically was 1379, and the minimum number was 1; the sum of records transferred was 223459.

**Fig.2:**
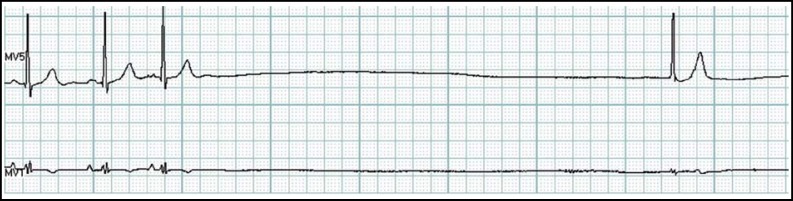
Sinus rhythm, sinus standstill (R-R intermission was 5.73 seconds), atrial premature beat, atrioventricular junctional escape beat accompanied by ST-segment depression

**Fig.3:**
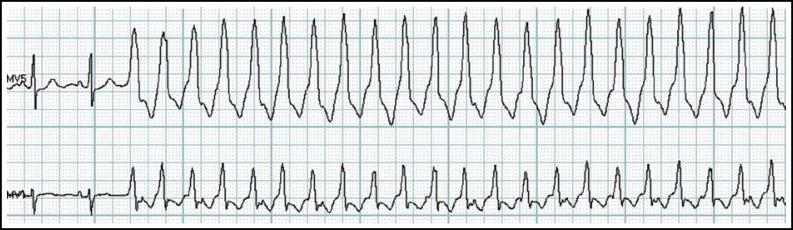
Sinus rhythm, sustained ventricular tachycardia.

Of the arrhythmias, the most frequent occurrence was atrial premature beat (6532 cases, 91.23%), followed by ventricular premature beat (5893 cases, 82.3%), superventricular tachycardia (1398 cases, 19.53%), atrial fibrillation (561 cases, 7.84%), ventricular tachycardia (374 cases, 5.23%) and various degrees of conduction block (197 cases, 2.75%), diminishingly. We discovered 274 cases of malignant arrhythmias, including 87 cases of serious sinus standstill (Fig.2) and 5 cases of sustained ventricular tachycardia (Fig.3), informed the patient immediately. Accordingly, many acute cardiac episodes were prevented because of the corresponding precautionary or therapeutic measures.

Of the ST-T changes, 2327 cases were diagnosed as coronary heart disease from a combination of symptoms and various examinations, whereas 939 cases needed further examinations. We discovered 43 cases of ST-segment elevation (suspicious of acute myocardial infarction) during monitoring sessions and advised to a nearby hospital at once. All cases were saved from danger, including 38 cases that received percutaneous coronary intervention. The time of SOTD, DTC, CTL, LTB and SOTB were compared with that of Yang Xinchun’s study via *t*-test respectively. The times of SOTD and SOTB had significant differences (*P*<0.05) between the two studies (Table-I).

## DISCUSSION

SCD is a major public health problem affecting 500,000 patients annually in the United States alone. The major risk factor for SCD is the presence of coronary artery disease, usually accompanied by a reduced ejection fraction. Globally, the incidence of SCD is expected to rise sharply as the prevalence of coronary artery disease and heart failure continue to increase.^18^ Furthermore, ventricular fibrillation (VF) and sustained ventricular tachycardia (VT) are also major causes of cardiac mortality, and both of these arrhythmias occur as a result of a complex interplay of abnormal substrates, myocardial vulnerability, imbalance of autonomic regulation, and so on. SCD is often the only manifestation of such malignant arrhythmias.^19^

a. Pre-warning device; b. Special mobile telephone; c. Leads’ line, d. Data line.

(1) The green electrode (V1) should be placed on the intersection of the right parasternal line and the fourth intercostal space, (2) the red electrode (V5) should be placed on the intersection of the left anterior axillary line and the fifth intercostal space, (3) the brown electrode (the mutual cathode) should be placed on the infraclavicular, (4) the black electrode (the outlying electrode) should be placed on the right chest.

**Table-I:** Comparison of the time of SOTD, DTC, CTL, LTB and SOTB with that of Yang Xinchun’s study

**Study’s**	**SOTD**	**DTC**	**CTL**	**LTB**	**SOTB**
Yang Xinchun’s study	183.2±150.1	3.5±2.1	30.6±11.1	24.8±10.2	277.7±159.3
Our study	55.5±25.4*	3.2±1.5	30.1±12.7	26.1±11.4	105.9±10.8*

Values are expressed as mean SD.

* *P*<0.05 vs. the Yang Xinchun’s study.

If remote cardiac monitoring of high-risk patients can give us early warning of major events and we can immediately implement the necessary interventions, we can greatly reduce both the mortality and cost of cardiovascular diseases. Hence, more attention is being paid to preventing acute cardiac episodes, such as cardiac rhythm problems and ST-T changes. Ambulatory Holter electrocardiography (ECG), noninvasive test to evaluate cardiac rhythm problems in various cardiac disorders, is widely used. Holter ECG can continuously examine cardiac rhythm throughout daily activities while permitting patient ambulatory activity. Long-term Holter ECG recording has an important role in predicting SCD by providing useful information about the frequency, rate and type of arrhythmias.^20^ However, Holter ECG can only be used to analyze electrocardiographic data retrospectively. TTM as a means for ambulatory arrhythmia detection and management of cardiac patients has demonstrated potential for considerable clinical utility. Because it is easy to use, relatively inexpensive and highly effective in the diagnosis of various cardiac disorders, it may be a preferred mode of surveillance for cardiac patients of all ages.^21^^-^^23^ TTM is superior to Holter ECG^24^, but it is also limited because it transfers electrocardiogram records only through immobile telephone.

In October 2004, the first cardiac remote monitoring center based on GPRS in Asia was founded at Qilu Hospital of Shandong University, and the cardiac remote monitoring system was applied to clinical practice formally. Its functions comprise continuous monitoring and pre-warning for electrocardiographic abnormalities as well as wireless and bidirectional transferring of electrocardiographic data through GPRS. Transfer modes involve automatic warning in addition to manual recording, and the patients can choose between them freely. When the mode of automatic warning is selected, the cardiac remote monitor worn by the patient can give an alarm while the electrocardiographic data is transferred to the cardiac remote monitoring center in the hospital through GPRS. This occurs in the instances of the heart rate going above 135 beats or under 45 beats per minute, ST-segment elevation or depression above 1mm, and a rest intermission above 2 seconds. This project since 2004 has completed nearly ten years, about 100,000 users were monitored, and accumulated a large number of ECG data. This is the only study of its kind in China, which makes our research unique. In this study, a number of cases of malignant arrhythmias or obvious ST-T changes were discovered, and they or their family members were informed by telephone at once; the corresponding precautionary or therapeutic measures were implemented so that the acute cardiac episodes were ended successfully. For the cases of obvious ST-T changes, the time of SOTD and SOTB were shorter significantly than in Yang Xinchun’s study (p<0.05). Early administration of reperfusion therapy could improve survival in patients with ST-elevation myocardial infarction by reestablishing coronary blood flow within the occluded infarct-related artery.^25^ A cardiac remote monitoring system could extend monitoring from in-hospital to out-of-hospital for patients who suffer from cardiovascular diseases and for sub-healthy people, and change the treatment strategy from urgent treatment after the acute cardiac episodes happen to monitoring the patients in real-time. As a result, patients’ quality of life could be improved, and the mortality and deformity rate of cardiovascular diseases, which seem to be increasing ceaselessly, could be reduced. It should be noted that, in China currently, there are many companies which have developed devices of Mobile remote cardiac monitoring. However, these devices are not used in clinical practice, which are all different from ours.

***Limitations of the study:*** Owing to only 2 leads on the remote monitor, our study was limited in evaluating inferolateral or high anterolateral myocardial infarction. Furthermore, there were occasions of false warning due to patients’ violent movements.

In conclusion, a cardiac remote monitoring system providing an automatic warning function can play an important role in preventing acute cardiac episodes and has favorable prospects for further development.

## Authors’ Contribution:

**CSQ and XSS** conceived, designed and did data collection &statistical analysis and wrote manuscript.

**GHQ** did review and final approval of manuscript.

**GHQ** also takes the responsibility and is accountable for all aspects of the work in ensuring that questions related to the accuracy or integrity of any part of the work are appropriately investigated and resolved.
